# Low fidelity simulation of small aortic annulus; understanding the relationship between anatomical structures and enlargement procedures

**DOI:** 10.1186/1749-8090-8-S1-P5

**Published:** 2013-09-11

**Authors:** A Hossien, I Khan, H Subhani, S Ashraf

**Affiliations:** 1 Cardiothoracic Surgery Department, Morriston Hospital, Swansea, UK

## Background

Aortic root occupies a central position in the fibrous skeleton with important relations to surrounding structures; therefore it is a challenge for surgeons enlarging the small annulus. We propose a low fidelity simulator to enhance the comprehension of the aortic root and the enlargement procedures.

## Methods

We used self-constructed models to simulate the aortic root. The related structures were constructed. Aortomitral angle of 120 degrees was created in the models. We performed three enlargement procedures 1. Manougian 2. Nicks 3. Nunez. Manougian and Nunez incisions were made through the commissure between the left and the Non coronary cusps (NCC) where Manougian extended through the anterior leaflet of the Mitral Valve (AML) while Nunez stopped proximal to the anterior annulus of the mitral valve. Nick’s incision was carried out through the middle of the NCC and also extending through the AML. Enlargements were carried out with a Dacron patch.

## Results

Self construction of the Aortic Root and its related structures results in improving of 3D understanding of their relationship. The creation of the aortomitral angle leads to understanding the importance of maintenance of this angle after enlargement. Manougian and Nicks procedures resulted in the opening of the left Atrium (LA) and subsequent repair of the LA roof in addition to closure of the Aortic Annulus. While Nunez was simpler to patch as it did not require additional repairs.

## Conclusion

Low fidelity simulator is an excellent tool in broadening the knowledge of the familiarizing and performing different types of aortic root enlargement.

